# Glycemic Variability Is Independently Associated With Poor Prognosis in Five Pediatric ICU Centers in Southwest China

**DOI:** 10.3389/fnut.2022.757982

**Published:** 2022-02-23

**Authors:** Milan Dong, Wenjun Liu, Yetao Luo, Jing Li, Bo Huang, Yingbo Zou, Fuyan Liu, Guoying Zhang, Ju Chen, Jianyu Jiang, Ling Duan, Daoxue Xiong, Hongmin Fu, Kai Yu

**Affiliations:** ^1^Department of Critical Care Medicine, Children's Hospital of Chongqing Medical University, National Clinical Research Center for Child Health and Disorders, Ministry of Education Key Laboratory of Child Development and Disorders, Chongqing, China; ^2^Department of Pediatrics, The People's Hospital of Yubei District of Chongqing City, Chongqing, China; ^3^Department of Clinical Epidemiology and Biostatistics, Children's Institute of Children's Hospital of Chongqing Medical University, Chongqing, China; ^4^Department of Pediatric Critical Care, The First People's Hospital of Zunyi, Zunyi, China; ^5^Department of Pediatric Critical Care, Chengdu Women's and Children's Central Hospital, Chengdu, China; ^6^Department of Pediatrics, Chongqing Three Gorges Women and Children's Hospital, Chongqing, China; ^7^Department of Pediatric Critical Care, Kunming Children's Hospital, Kunming, China

**Keywords:** dysglycemia, glycemic variability, MAG, pediatrics, critically ill, mortality

## Abstract

**Background:**

Glucose variability (GV) is a common complication of dysglycemia in critically ill patients. However, there are few studies on the role of GV in the prognosis of pediatric patients, and there is no consensus on the appropriate method for GV measurement. The objective of this study was to determine the “optimal” index of GV in non-diabetic critically ill children in a prospective multicenter cohort observational study. Also, we aimed to confirm the potential association between GV and unfavorable outcomes and whether this association persists after controlling for hypoglycemia or hyperglycemia.

**Materials and Methods:**

Blood glucose values were recorded for the first 72 h and were used to calculate the GV for each participant. Four different metrics [SD, glycemic lability index (GLI), mean absolute glucose (MAG), and absolute change of percentage (ACACP)] were considered and compared to identify the “best” GV index associated with poor prognosis in non-diabetic critically ill children. Among the four metrics, the SD was most commonly used in previous studies, while GLI- and MAG-integrated temporal information, that is the rate and magnitude of change and the time interval between glucose measurements. The fourth metric, the average consecutive ACACP, was introduced in our study, which can be used in real-time clinical decisions. The primary outcome of this study was the 28-day mortality. The receiver operating characteristic (ROC) curve analysis was conducted to compare the predictive power of different metrics of GV for the primary outcome. The GV index with the largest area under ROC curve (AUC) was chosen for subsequent multivariate analyses. Multivariate Cox regression analysis was performed to identify the potential predictors of the outcome. To compare the contribution in 28-day mortality prognosis between glycemic variability and hyper- or hypoglycemia, performance metrics were calculated, which included AUC, net reclassification improvement (NRI), and integrated discrimination improvement (IDI).

**Results:**

Among 780 participants, 12.4% (*n* = 97) died within 28 days after admission to the pediatric intensive care unit (PICU). Statistically significant differences were found between survivors and non-survivors in terms of four GV metrics (SD, GLI, MAG, and ACACP), in which MAG (*AUC*: 0.762, 95% CI: 0.705–0.819, *p* < 0.001) achieved the largest AUC and showed a strong independent association with ICU mortality. Subsequent addition of MAG to the multivariate Cox model for hyperglycemia resulted in further quantitative evolution of the model statistics (*AUC* = 0.651–0.681, *p* = 0.001; *IDI*: 0.017, *p* = 0.044; *NRI*: 0.224, *p* = 0.186). The impact of hyperglycemia (adjusted hazard ratio [*aHR*]: 1.419, 95% *CI*: 0.815–2.471, *p* = 0.216) on outcome was attenuated and no longer statistically relevant after adjustment for MAG (*aHR*: 2.455, 95% *CI*: 1.411–4.270, *p* = 0.001).

**Conclusions:**

GV is strongly associated with poor prognosis independent of mean glucose level, demonstrating more predictive power compared with hypoglycemia and hyperglycemia after adjusting for confounding factors. GV metrics that contain information, such as time and rate of change, are the focus of future research; thus, the MAG may be a good choice. The findings of this study emphasize the crucial role of GVs in children in the PICU. Clinicians should pay more attention to GV for clinical glucose management.

## Introduction

Glucose management is an important element of intensive care management. In general, glucose dysregulation is related to negative outcomes in critically ill patients and is a multi-system complication (1). A few authors have explored the effectiveness of glucose levels on the prognosis of glucose dysregulation with different glucose management protocols; however, the results are contradictory, especially regarding the question of whether strict control of glucose levels is linked to improve clinical prognosis (2, 3). Increased glucose variability (GV) increases the risk of poor outcomes, which may explain the contradictory results in the literature (4).

In 2006, Egi et al. (5) demonstrated for the first time that GV is closely related to poor outcomes in critically ill adults. In recent years, a growing number of studies have confirmed that GV is independently associated with unfavorable outcomes in critically ill adults, regardless of its definition (6–10). However, fewer studies, which explored the impact of GV on negative prognosis have focused on pediatric populations, compared to adults, and most of the previous studies (11, 12) were retrospectively conducted in a single center. Thus, prospective multicenter studies are needed on the association between adverse prognosis and GV in pediatric patients. Subsequently, we conducted this study with a prospective analysis of a pediatric cohort to address this clinical issue.

The GV, also known as glucose fluctuation, represents the fluctuation of glucose levels over time and refers to a non-stationary state in which glucose levels fluctuate between high and low values, such as speed, magnitude, and frequency. A few GV metrics appear to be correlated with worse outcomes in critically ill patients, such as standard deviation (7, 8, 13), coefficient of variation (7, 13, 14), mean amplitude of glycemic excursion (7, 13, 15), mean absolute glucose (13, 16), and glycemic lability index (GLI) (15). These measurements differ in the requirements for calculations, such as the frequency or time interval between glucose measurements. Nevertheless, there is currently no consensus on the definition of GV and the “best” index.

Our retrospective study (17) also demonstrated that GV was independently associated with unfavorable outcomes. In addition to GV, hyperglycemia and hypoglycemia have been previously reported to be associated with poor prognosis in pediatric patients (11, 18–20). Therefore, in this study, we prospectively investigated whether the correlation between GV and poor prognosis remained after controlling for hyperglycemia and hypoglycemia. This study aimed to broaden the understanding of the ways in which GV, hypoglycemia, and hyperglycemia are associated with adverse clinical outcomes in children admitted to the pediatric intensive care units (PICUs) and to improve our understanding of glucose management.

## Materials and Methods

### Study Design and Quality Control

This was a prospective, multicenter, observational study conducted between January 1, 2020 and December 31, 2020. The participants were recruited from five PICUs that belong to tertiary A hospitals in southwest China and represent the top intensive care centers, providing top-tier medical care to most critically ill children in these regions. The ethical committee of the Children's Hospital of Chongqing Medical University approved this study (File No. 2019,37), and the clinical trial registration was completed (ChiCTR2000030846).

Based on the literature, a sample size of 665 can produce 90% power at the 5% level of significance when considering a 20% lost-to-review rate, which was calculated based on the primary outcome-28-day mortality in PICU with cohort study analysis using an estimated intensive care unit (ICU) mortality of 10% and a risk ratio of 2 (21, 22).

Before initiating the study, we provided professional training for data loggers in each research PICU, including project objectives, research progress, and data collection. Multi-center data collection was performed by recorders under strict guidelines. After data collection and entry, the data were sent to a central repository where they were corrected for any suspicious errors or missing values by the chief coordinator and physicians.

### Inclusion and Exclusion Criteria

All critically ill children aged 36 weeks (corrected gestational age) to 16 years who were receiving vasoactive drug support for hypotension or ventilatory support for respiratory failure was considered for inclusion. Exclusion criteria included the following children: (1) those who remained in the PICU for <24 h, (2) those who were diagnosed with diabetes mellitus or inborn errors of metabolism, (3) those who had utilized glucocorticoids or total parenteral nutrition (TPN) treatment for 3 days after PICU admission; (4) those who were readmitted to the PICU.

### Data Collection

Data were manually entered into the Epidata software, version 3.1. For each patient, demographic data (age, sex, and nutritional status), clinical treatments [vasoactive and glucose infusions, mechanical ventilation (MV), and other adjuvant therapies], and prognosis (28-day mortality, multi-organ dysfunction syndrome (MODS), and length of time in the PICU) were recorded. The primary outcome was 28-day mortality. Secondary outcomes were the number of days alive and free from MV at 28 days (ICU free days to day 28, MV free days to day 28) after admission. If the patient died within 28 days, we recorded a 0. If a patient survived for a period within 28 days, we recorded 28 minus the survival time; the smaller this value, the worse the prognosis, which is similar to the record of MV free days to day 28. In addition, the incidence of MODS was recorded as a poor prognosis indicator. The follow-up for each patient admitted to our PICUs was performed and recorded by the nurse 1 month after discharge using telephone interviews (the questionnaire is shown in Supplementary Material). We chose the Pediatric Index of Mortality-2 (PIM2) score (23) to estimate the severity of the illness at the time of admission to the PICUs. The index was used to determine nutritional status as the “z” score for weight/height (≤ 5 years old) or BMI (>5 years old) for age using the Epi-Info software. Overweight was defined as BMI for age > +2 SD, and wasting was <-2SD.

### Blood Glucose Indices

We collected all blood glucose values for the first 72 h after admission. The children in the PICU were examined for blood gases at least twice per day based on their daily treatment. Consequently, no additional blood specimens were performed. The specimens were mainly from arterial blood but also included venous and capillary blood sources.

For each patient, we calculated the following metrics (the reasons for GV metrics included in our study are shown in Supplementary Material) based on all the available glucose values: the average glucose (Mean), *SD*, GLI [*GLI* = ∑(ΔBG^2^/Δh)]/d, (mmol/L)2/h/d) (7), and mean absolute glucose (MAG) [MAG = (∑Δ BG)/Δh, (mmol/l)] (16). In addition, the average consecutive absolute change percentage [ACACP = 1/(ni-1) * ∑(|Xi−Xi−1|/Xi), %] (24) was introduced. Indeed, an index can be conveniently used in real-time to aid with clinical decisions. Glucose measurement upon admission (Adm) was the first glucose value after ICU admission. We chose to define hypoglycemia as a glucose value ≤ 3.6 mmol/L and hyperglycemia cut-off values of 8.3 mmol/L based on both adult and pediatric studies (11, 25).

### Blood Glucose Management

As per the existing unit policy, dextrose infusion is stopped when the glucose value exceeds 15 mmol/L and actively deals with the primary disease (26). Then, we observe for 1 h: if there was no downward trend (glucose decreased to <4 mmol/L/h), we could infuse insulin (0.05–0.1 U/kg). Insulin infusion was stopped when the blood glucose level was below 10 mmol/L (27, 28).

### Statistical Analysis

Statistical analyses were performed using the R version 4.0.0. Categorical variables were described by *n* (%), and continuous variables without normal distribution were described by the median (IQR). For comparisons between the two groups, the Wilcoxon rank-sum test was used for continuous variables without normal distribution, while the chi-square test was used for categorical variables. We used ROC curve analysis to compare the predictive power of different indicators of GV for the primary outcome. The GV index with the largest area under ROC curve (AUC) was also converted to a categorical variable based on the cut-off value for subsequent analysis. The GV index and hypo/hyperglycemia were gradually substituted into a multivariate Cox regression model adjusted for confounders with a significance level of *p* ≤ 0.10 to prevent the exclusion of significant variables from the regression model. The results are expressed as hazard ratio (HR) (95% CI). The overall predictive accuracy of the model was quantified using the AUC. In addition, net reclassification improvement (NRI) and integrated discrimination improvement (IDI) analyses (29) were performed to assess GV and the predictive power of hypoglycemia/hyperglycemia. Depending on the type of outcome variable, multivariable logistic regression or linear regression can also be used to assess the impact of GV on other outcome indicators (e.g., ICU-free days or ventilator-free days to day 28, and MODs). A value of *p* < 0.05 for a two-sided test is considered statistically significant.

## Results

**Figure 1:**
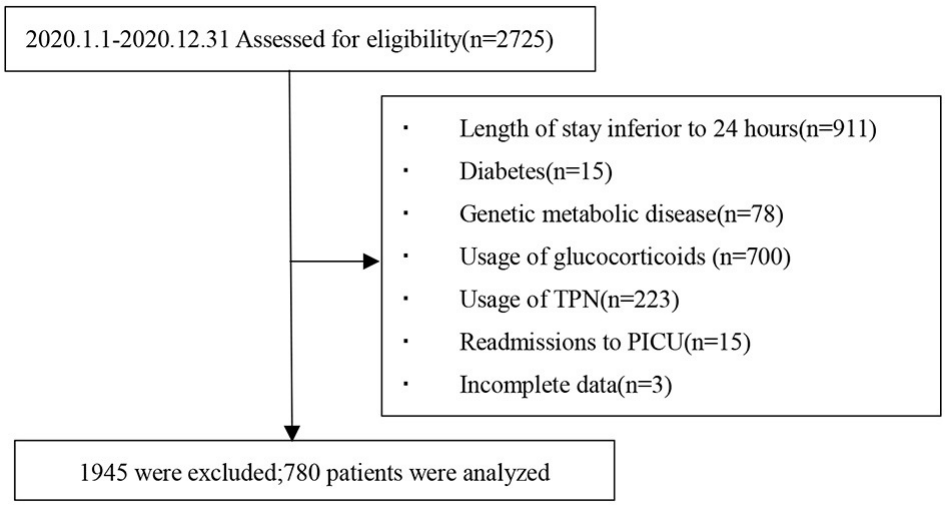
Flowchart illustrating the inclusion and exclusion of participants for the study.

We consecutively monitored a total of 2,725 patients in the five participating units during the study period in which 780 patients met the nadir criteria for entry into the study. A flowchart of the case screening is shown in Figure 1.

In total, 97 (12.4%) of the 780 participants died within the 28-day period after PICU admission. At the primary endpoint (28-day mortality), participants were divided into survival and non-survival groups. The primary reasons for ICU admission included 353 cases of respiratory diseases (such as severe pneumonia and asthma), 35 cases of cardiovascular diseases (such asshock), 130 cases of neurological diseases, 85 cases of trauma, 14 cases of postoperative care, 58 cases of sepsis, and 105 other cases (such as oncologic, renal, metabolic, and hematologic cases). A total of 9,142 glucose values were collected of which 65.9% were from arterial blood gas analysis, 3.4% from venous blood, and 30.7% from capillary blood, with a median frequency of 11 (IQR: 8.25–14) glucose monitoring per patient. Demographic information, clinical features, and disease severity between the survival and non-survival groups are shown in Table 1.

**Table 1:** The comparison of baseline variables between survivors and non-survivors[M (P25, P75), *n*(%)].

	**All (*n* = 780)**	**Survivors (*n* = 683)**	**Non-survivors (*n* = 97)**	**X^**2**^/Z**	***P* value**
**Age group/*****n*** **(%)**					
<3ys	542.(69.5)	483.(70.7)	59.(60.8)		
3ys to <16ys	238 (30.5)	200 (29.3)	38 (39.2)	3.920	0.059
Male/*n* (%)	464 (59.5)	404 (59.2)	60 (61.9)	0.258	0.659
Medical/*n* (%)	600 (76.9)	522 (76.4)	78 (80.4)	0.760	0.441
**BMZ/*****n*** **(%)**					
< -2	173 (22.2)	149 (21.8)	24 (24.7)		
−2~2	551 (70.6)	481 (70.4)	70 (72.2)		
>2	56 (7.2)	53 (7.8)	3 (3.1)	2.941	0.232
MV/*n* (%)	588 (75.4)	495 (72.5)	93 (95.9)	25.068	<0.001
CRRT/*n* (%)	51 (6.5)	29 (4.2)	22 (22.7)	47.233	<0.001
CPR/*n* (%)	26 (3.3)	11 (1.6)	15 (15.5)	–	<0.001
Vasoactive drug/*n* (%)	182 (23.3)	126 (18.4)	56 (57.7)	73.273	<0.001
Insulin/*n* (%)	27 (3.5)	10 (1.5)	17 (17.5)	–	<0.001
GIR	0.7 (0.4,1.2)	0.6 (0.3,1.1)	1.0 (0.5,1.7)	−4.793	<0.001
PIM2 score	−2.6 (−3.3, −1.9)	−2.6 (−3.5, −2.1)	−1.4 (−2.6, 1.9)	−7.240	<0.001

Table 2 illustrates the comparative differences in glucose metrics between the survival and non-survival groups, showing that all glucose metrics were significantly lower in the survivors group than in the non-survivors group (*p* < 0.05).

**Table 2:** The comparison of glucose metrics between survivors and non-survivors [M (P25, P75), *n*(%)].

**Metrics**	**All (*n* = 780)**	**Survivors (*n* = 683)**	**Non-survivors (*n* = 97)**	**c^**2**^/Z**	***P* value**
Adm	5.9 (5.1, 7.2)	5.8 (5.0, 7.1)	6.6 (5.2, 9.6)	−3.094	0.002
Mean	5.8 (5.4, 6.4)	5.8 (5.4, 6.3)	6.2 (5.3, 7.9)	−2.878	0.004
Hyperglycemia/n (%)	289 (37.1)	224 (32.8)	65 (67.0)	42.630	<0.001
Hypoglycemia/n (%)	155 (19.9)	119 (17.4)	36 (37.1)	20.681	<0.001
SD	1.0 (0.7, 1.5)	0.9 (0.7, 1.4)	1.6 (1.1, 3.0)	−7.279	<0.001
GLI	1.6 (0.6, 4.4)	1.4 (0.6, 3.6)	6.1 (1.7, 43.6)	−7.316	<0.001
MAG	0.2 (0.1, 0.3)	0.1 (0.1, 0.2)	0.3 (0.2, 0.8)	−8.360	<0.001
ACACP (%)	16.9 (12.8, 23.0)	16.5 (12.5, 22.2)	22.3 (16.4, 32.6)	−5.788	<0.001

The AUCs are shown in Table 3, which were plotted to evaluate the ability of glucose metrics and PIM2 score in predicting 28-day PICU mortality. These results separately correlated with PICU 28-day mortality (*AUC* > 0.700, *p* < 0.05). However, MAG (*AUC*: 0.762, 95% *CI*: 0.705–0.819, *p* < 0.001) has the largest areas compared to other GV indices in the prediction of PICU 28-day mortality. MAG had significantly better discrimination of PICU 28-day mortality than the other metrics (*p* < 0.05). Therefore, we used MAG to represent GV and explore its impact on adverse outcomes. Additionally, we utilized an optimal cut-off value for 28-day mortality prediction.

**Table 3:** Receiver operating characteristic curves for mortality on critically ill children.

	**AUC (95%CI)**	**Cutoff value (sensitivity/specificity, %)**	***P*** 值	**+LR**	**–LR**	**+PV**	**–PV**
Adm	0.597 (0.530, 0.664)	6.70 (48.45, 71.89)	0.002	1.72	0.72	19.7	90.8
PIM2	0.727 (0.668, 0.786)	−1.88 (56.70, 81.55)	<0.001	3.07	0.53	30.4	93.0
Mean	0.590 (0.518, 0.662)	7.03 (32.99, 90.48)	0.004	3.47	0.74	33.0	90.5
SD	0.728 (0.671, 0.785)	1.11 (75.26, 62.23)	<0.001	1.99	0.40	22.1	94.7
GLI	0.729 (0.670, 0.789)	4.37 (57.73, 79.65)	<0.001	2.84	0.53	28.7	93.0
MAG	0.762 (0.705, 0.819)	0.27 (59.79, 82.58)	<0.001	3.43	0.49	32.8	93.5
ACACP	0.681 (0.621, 0.742)	24.20 (47.42, 81.55)	<0.001	2.57	0.64	26.7	91.6

We sequentially included the baseline variables in the Cox regression model with *p* ≤ 0.1 in Table 1, followed by hypoglycemia and MAG, respectively. The results of the multivariate regression model showed that hypoglycemia was not associated with 28-day mortality (Table 4, Model 2) after adjusting for significant confounders. However, GV represented by MAG (adjusted *HR*: 2.795, 95% *CI*: 1.687–4.632, *p* < 0.001) was still related to PICU mortality after adjustment for hypoglycemia (Table 4, Model 3). The addition of MAG to the model for hypoglycemia led to further quantitative evolution of the model statistics (*AUC*: from 0.616 to 0.668, *p* < 0.001; *IDI*: 0.030, *p* = 0.006; *NRI*: 0.325, *p* = 0.022). There was no interaction between MAG and hypoglycemia.

**Table 4:** Multivariable COX regression: associations of hypoglycemia and MAG With 28-day mortality.

**Sequential models for 28-day mortality**	**Main variables**	**HR (95%CI)**	***P* value**	**Model statistics**		
				**Likelihood ratio** **chi-square**	***P*^‡^ *value***	**AUC** **(95%CI)**
Multivariable model 1:	Mean	1.049 (0.928, 1.187)	0.443	290.04	–	0.607 (0.541, 0.674)
Multivariable model 2: model1+hypoglycemia	Mean	1.065 (0.942, 1.205)	0.313	296.739	0.168	0.616 (0.552, 0.681)
	Hypoglycemia	1.415 (0.870, 2.300)	0.162			
Multivariable model3: model2+MAG	Mean	0.974 (0.853, 1.112)	0.689	304.543	<0.001	0.668 (0.608, 0.729)
	Hypoglycemia	1.195 (0.735, 1.942)	0.472			
	MAG	2.795 (1.687, 4.632)	<0.001			

*Model 1: adjusted for confounders with a significance level of P ≤ 0.10 from Table 1, including PIM2 score, Mean, and so on. Model 2: adjusted for factors as in model 1, and hypoglycemia. Model 3: adjusted for factors as in model 2, and MAG*.

‡ *For likelihood ratio test between sequential models; the P-value is for comparison of the multivariable model to the previous model*.

We constructed the same model described above by replacing hypoglycemia with hyperglycemia. After adjustment for confounding factors, patients with hyperglycemia experienced increased rates of 28-day mortality (adjusted *HR* for hyperglycemia, 2.026; 95% *CI*: 1.240–3.310; *p* = 0.005) (Table 5, Model 2). Subsequent addition of MAG to the model for hyperglycemia resulted in an early incremental improvement in model statistics (*AUC*: from 0.651 to 0.681, *p* = 0.001; *IDI*: 0.017, *p* = 0.044; *NRI*: 0.224, *p* = 0.186). After adjusting for MAG (adjusted *HR*: 2.455, 95% *CI*: 1.411–4.270, *p* = 0.001), the effect of hyperglycemia (adjusted *HR*: 1.419, 95% *CI*: 0.815–2.471, *p* = 0.216) (Table 5, Model 3) on the adverse outcome was attenuated and no longer statistically relevant. In the final model, MAG had a greater prognostic contribution than hyperglycemia (χ^2^*-df* (30): 9.115 and 0.528 for MAG and hyperglycemia, respectively).

**Table 5:** Multivariable COX regression: associations of hyperglycemia and MAG With 28-day mortality.

**Sequential models for 28-day mortality**	**Main variables**	**HR (95%CI)**	***P value***	**Model statistics**		
				**Likelihood** **ratio** **chi-square**	***P*^‡^ *value***	**AUC**
Multivariable model 1:	Mean	1.049 (0.928, 1.187)	0.443	290.04	–	0.607 (0.541, 0.674)
Multivariable model 2: Model 1+Hyperglycemia	Mean	0.980 (0.860, 1.117)	0.765	290.155	0.005	0.651 (0.590, 0.712)
	hyperglycemia	2.026 (1.240, 3.310)	0.005			
Multivariable model 3: Model 2+MAG	Mean	0.942 (0.824, 1.075)	0.385	303.479	0.001	0.681 (0.622, 0.739)
	Hyperglycemia	1.419 (0.815, 2.471)	0.216			
	MAG	2.455 (1.411, 4.270)	0.001			

*Model 1: adjusted for confounders with a significance level of P ≤ 0.10 from Table 1, including PIM2 score, Mean, and so on. Model 2: adjusted for factors as in model 1, and hyperglycemia. Model 3: adjusted for factors as in model 2, and MAG*.

‡ *For likelihood ratio test between sequential models; the P-value is for comparison of the multivariable model to the previous model*.

**Figure 2:**
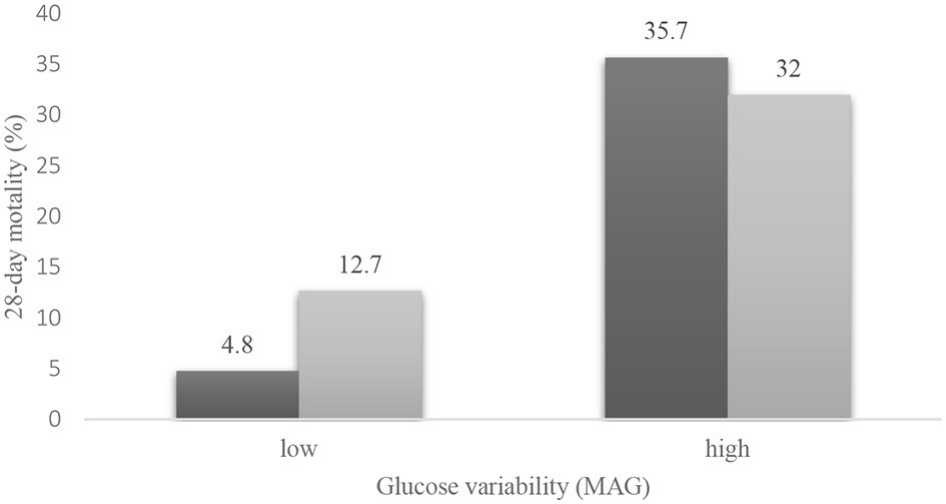
Mortality for hyperglycemia and glucose variability.

However, the interaction between hyperglycemia and MAG was statistically significant (*p* = 0.016). We used Cochran's and Mantel–Haenszel statistics to further explore the relationship between hyperglycemia, MAG, and mortality (Figure 2). Higher mortality was observed in the high GV group than in the low GV group (*p* < 0.001), regardless of the presence of hyperglycemia. The test of homogeneity of the odds ratio (OR) was *p* = 0.022; therefore, after stratifying by MAG, in the low GV group, hyperglycemia was relevant to PICU mortality (*OR* = 2.910, 95% *CI*: 1.513–5.596, *p* = 0.001). In the high GV group, hyperglycemia did not affect mortality (*OR* = 0.846; 95% *CI*: 0.363–1.974, *p* = 0.699). This means that patients with hyperglycemia will have different mortality rates depending on the MAG. In contrast, in the high GV group, hyperglycemia did not play a role in mortality events. These results support the possibility that GV may be a stronger predictor of 28-day mortality than hyperglycemia.

Table 6 shows the effect of blood fluctuations on ICU-free days to day 28, MVfree days to day 28, and the incidence of MODSs. The results show that the median ICU-free days in the high variability group were lower than that of the low group (15 [0.0–22.9] vs. 22 [15.1–24.5], *p* = 0.001). Similar results were observed in the ventilator-free days (21.5 [0.0–26.1] vs. 25.7 [21.0–28.0], *p* < 0.001). The incidence of MODs was significantly higher in the high-GV group than in the low-GV group (62.9 vs. 29.5%, *p* < 0.001).

**Table 6:** Effect of Glucose variability on 28-day ICU-free days, 28-day ventilator-free days, and multiorgan dysfunction syndrome [M(IQR)/*n* (%)].

	**MAG (mmol/L)**		***P value***
	**<0.27**	**≥0.27**	
28-day ICU-free days	22.0 (15.1, 24.5)	15 (0.0, 22.9)	0.001
28-day ventilator-free days	25.7 (21.0, 28.0)	21.5 (0.0, 26.1)	<0.001
Multiorgan dysfunction syndrome	176 (29.1)	110 (62.9)	<0.001

*Adjusted for factors with a significance level of P ≤ 0.10 from Table 1*.

## Discussion

To our knowledge, this is the first multicenter prospective study of pediatric dysglycemia (such as hypoglycemia, hyperglycemia, and GV) conducted in Southwest China, which attempted to establish an association between dysglycemia and 28-day mortality in PICUs. Several important relationships were also observed. First, GV was independently associated with unfavorable outcomes, and it may play a more predictive role in PICU mortality than hyperglycemia and hypoglycemia. These findings emphasize the important role of GV in children admitted to the PICU. Also, MAG may be a dominant index reflecting glucose fluctuations, which considers the speed and magnitude of change, and the time series among glucose values.

A strong relationship between increasing GV and poor prognosis has been consistently reported in adult patients (6–10, 31). Few studies in this field have been conducted in pediatrics; however, similar insights have been obtained. In 2006, Wintergerst et al. (11) retrospectively observed that increased GV was related to increased hospitalization stay and mortality rates in 1,094 PICU admissions in a single center; however, the severity of the disease was ignored. Hirshberg et al. (22) performed a retrospective study of 863 non-diabetic children in the PICU during a 1-year period and found that the incidence of glucose fluctuations (defined as having both hyperglycemia and hypoglycemia) was 6.8%, and glucose fluctuations were still associated with high mortality after adjusting for PRISM score. In another retrospective cohort of 101 critically ill children, researchers found that increased GV was associated with increased mortality (12). Similarly, Bhutia et al. (21) prospectively observed 170 critically ill children and found that GV was related to multi-organ dysfunction. Pinchefsky et al. (32) found that GV was independently associated with the deterioration of brain function in neonates with encephalopathy. Although these studies involved children in a single clinical setting, they add to the evidence that GV is associated with adverse outcomes. In our series, we further prospectively investigated the relationship between GV and deleterious effects in multiple centers, and similar results were reported in our findings. Based on our results, GV was associated with mortality, MODs, and ventilator/ICU-free days, which confirmed the strong predictive role of GV in children in the PICU. This association may be attributed to oscillatory glucose induction that exacerbates oxidative stress, promotes inflammatory responses, and regulates ROS-mediated NFκB/RAGE activation, resulting in endothelial cell injury (33–35). Taken together, these results emphasize the dangerous role of GV in critically ill patients. Indeed, management of GV may be warranted in glucose management regimens.

Our data are also complementary to findings from observational studies that evaluated the association between hypo/hyperglycemia and GV during critical illness and mortality. The results of our study suggest that GV may be a more powerful independent risk factor of mortality in patients in the PICU than hyperglycemia and hypoglycemia. Consistent with our findings, Bagshaw et al. (36) found that glucose fluctuation, defined as having both hypoglycemia and hyperglycemia, was relevant to a larger *OR* of ICU (1.5 vs. 1.2 vs. 1.0, *p* < 0.05) and hospital (1.4 vs. 1.2 vs. 1.0, *p* < 0.05) mortality, compared to only hypoglycemia or hyperglycemia, or neither. Several *in vitro* or animal experiments (37–39) have demonstrated that oscillating glucose levels aggravate oxidative stress compared to persistent hyperglycemia, resulting in accelerated cell injury and apoptosis. Quagliaro et al. (37) demonstrated that in umbilical vein cells, the levels of protein kinase C-β were higher in the rapid glucose fluctuation group than in the sustained hyperglycemia group.

From the perspective of clinical treatment, a single blood glucose value can be influenced by various factors, such as medication, diet, and stress status. Therefore, hyperglycemia or hypoglycemia determined by a single value may not accurately reflect the metabolic status of critically ill patients. In contrast, GV is calculated using a few glucose values and may reveal dynamic glucose changes. This may be one explanation that glucose fluctuation may be a stronger predictor of mortality than hyperglycemia and hypoglycemia. Therefore, GV should be considered as an important predictor in future studies of prognostic models in critically ill patients, and clinicians should pay more attention to dynamic fluctuations of glucose versus single glucose values.

In contrast to the previous study, which only used a single index that defined GV, our study selected four indicators (SD, GLI, MAG, and ACACP). ROC analysis showed that three of the four GV indicators (SD, GLI, and MAG) had a moderate predicted value for 28-day PICU mortality (AUC > 0.7, *p* < 0.05). Among all four indices, MAG obtained the largest AUC and was found to be a better indicator of GV than SD. Hermanides et al. (16) illustrated that the glucose profile demonstrated different MAGs even in the presence of the same SD value, and MAG was superior to SD in terms of predicting mortality. Variability should be considered in terms of the speed and magnitude of change, as well as the time interval between glucose measurements; meanwhile MAG does account for this variability, but *SD* does not. Our retrospective study (17) found that GLI, a variability indicator that also accounts for the arrangement of estimations and time, had increased predictive power for PICU mortality compared to SD, CV, and MAGE (*AUC*: 0.626 vs. 0.601, 0.599, 0.573). Based on the results of previous studies (7, 15) and our own results, GV index which accounts for the range, speed, time series, should be used; MAG, or GLI may be a good choice. Given its clinical practicality, we introduced ACACP as a GV indicator, which is simple to apply to a treatment protocol (24). This index focused on the comparison of changes in subsequent blood glucose values. Although the AUC of ACACP was smaller than that of MAG in our dataset, its unique calculation and clinical significance suggest that GV metrics should reflect the change from one value to the next and guide clinical management to minimize this change, thereby facilitating glycemic management and improving prognosis in clinical practice. Finding indicators that contain information, such as time series, rate, and range of change, and that are convenient for clinical application is a potential practical target for future treatment.

The American College of Critical Care Medicine suggested that a target BG that is in the range of 100–180 mg/dL may be a reasonable goal for critically ill pediatric patients (40). In Surviving Sepsis Campaign international guidelines, there is a consensus to target glucose levels below 180 mg/dL (10 mmol/L) for children with septic shock (41). These guidelines focus only on the level of absolute glucose values. The Clinical Practice Guideline (40) states that GV needs to be used as an indicator of clinical glucose control management. Our findings also suggest that glucose management should not only consider the glucose value, but also GV. The available literature was inadequate to support recommendations regarding GV measurements and standard indices in pediatric patients. The glucose regimen and monitoring system should be designed to minimize glycemic variability in clinical practice. Tracking and minimizing differences from one blood glucose measurement to the next is a potential practical target for future treatment. ACACP as a practical indicator, combined with continuous glucose monitoring (CGM), will likely increase the safety and acceptance of GV management protocols. CGM may be a good choice for practical, real-time treatment goals, regardless of its high price and requirements for use.

The multicenter, prospective design is a major strength of this study, particularly the choice to assess four GV indices in a large cohort of unselected children in the PICU and to explore their relationship with adverse outcomes. The results have a wider applicability than those of single-center studies. In addition, GV in the early stages may reflect a physiological stress response; indeed, as treatment duration increases, it may be influenced by many factors related to treatment, such as nutritional intake and drug usage (42). In the present study, GV metric calculations included the first 72 h of glucose values and excluded children using glucocorticoids or TPN, reducing the impact of medical management. Thus, GV in our study may predict mortality. However, our data on glucose included only the first 72 h after admission to the PICU, which represents a short period; thus, there may be a bias in the results. However, our large cohort may have eliminated this effect to some extent.

Our study has some limitations. First, there may be ascertainment bias, as patients with abnormal blood glucose levels may have more frequent measurements, while those with normal blood glucose levels have a comparatively reduced number of measurements. As a result, we may miss the record of hyperglycemia or hypoglycemia in some patients. Ideally, CGM should be performed at predetermined time points; however, this will increase the amount of blood sampled and, thereby, the level of discomfort experienced by young children. Advances in CGM technology will facilitate the management or monitoring of early GV in critically ill children. In addition, our glucose measurements were obtained from different blood specimens, and homogeneity could not be ensured. Finally, our study cannot draw conclusions about causality, since the results of observational studies can only assume that an association exists.

## Conclusions

Our results endorse and expand on previous literature findings by showing that fluctuations in blood glucose control negatively affect clinical prognosis in children in the PICU, and GV may be more strongly associated with mortality outcomes compared to hypoglycemia and hyperglycemia. Clinicians in PICUs should pay more attention to GV. MAG may be the dominant indicator of GV, which contains information on the speed and magnitude of blood glucose changes. These results emphasize the importance of monitoring GVs in children in the PICU, such as the use of the CGM system. Exploring the mechanisms of GV and optimizing personal glycemic management in PICUs will be the focus of future research to minimize the potential risk of healthcare-acquired events.

## Data Availability Statement

The original contributions presented in the study are included in the article/Supplementary Material, further inquiries can be directed to the corresponding author/s.

## Ethics Statement

Written informed consent was obtained from the individual(s), and minor(s)' legal guardian/next of kin, for the publication of any potentially identifiable images or data included in this article.

## Author Contributions

JL participated in the study design, project management, and supervision. MD and WL were responsible for the conceptualization and design of forms, data management and quality control, writing, and editing. JL, BH, YZ, GZ, JJ, and HF were responsible for the project implementation organization and management and data quality control. MD and YL were responsible for the statistical analysis and documentation. MD, WL, FL, JC, LD, DX, and KY were responsible for the recruitment of participants and documentation of data. All authors were contributed to this article and approved the submitted version.

## Funding

This study was supported by the General Project Fund for National Clinical Research Center for Child Health and Disorders (grant number: NCRC-2019-GP-12).

## Conflict of Interest

The authors declare that the research was conducted in the absence of any commercial or financial relationships that could be construed as a potential conflict of interest.

## Publisher's Note

All claims expressed in this article are solely those of the authors and do not necessarily represent those of their affiliated organizations, or those of the publisher, the editors and the reviewers. Any product that may be evaluated in this article, or claim that may be made by its manufacturer, is not guaranteed or endorsed by the publisher.

## Abbreviations

ACACP: Average consecutive absolute change percentage
Adm: Blood glucose concentration at admission
AUC: Area under the curve
BAZ: Body mass index for age Z score
BMI: Body mass index
CPR: Cardiopulmonary resuscitation
CRRT: Continuous renal replacement therapy
GIR: Glucose infusion rate
GLI: Glycemic lability index
HR: Hazard Ratio
MAG: Mean absolute glucose
Mean: Average blood glucose
MODS: Multi-organ dysfunction syndrome
MV: Mechanical ventilation
PICU: Pediatric intensive care unit
ROC: The receiver operating characteristic
TGC: Tight glycemic control
PIM2: Pediatric index of mortality score II
+LR: Positive likelihood ratio
–LR: Negative likelihood ratio
+PV: Positive predictive value
–PV: Negative predictive value
IQR: Interquartile range
ROS: Reactive oxygen species
NFκB/RAGE: The nuclear factor-kappa B/Receptor for advanced glycation end products.
