# Supplementary material for: Glycemic Variability Is Independently Associated With Poor Prognosis in Five Pediatric ICU Centers in Southwest China
Source: Front Nutr. 2022 Feb 23;9:757982. doi: 10.3389/fnut.2022.757982 (PMC8905539; doi:10.3389/fnut.2022.757982)
Supplement: Supplementary file 1 [file Data_Sheet_1.pdf]

the follow-up forms of PICU

1.The current condition of the children who have been discharged (comment)

○ [ ]

○ Dead children, not involved

2. the condition after discharged（comment）

○ Not treatment

○ Rehabilitation in our hospital

○ Continue treatment at the local hospital

○ Continue treatment in a better hospital

○ Dead children, not involved

3. What else help is needed now（comment)

○ Basic nursing

○ Medication guidance

Rehabilitation at home

○ Psychological help

○ Fertility guidance

○ Medical insurance reimbursement

○ The inspection results

○ Needn't any help

4. Satisfaction with our work, comments and Suggestions（comment）

○ Great satisfaction

○ Satisfaction

○ General satisfaction

○ Dissatisfaction

confirm　　eliminate

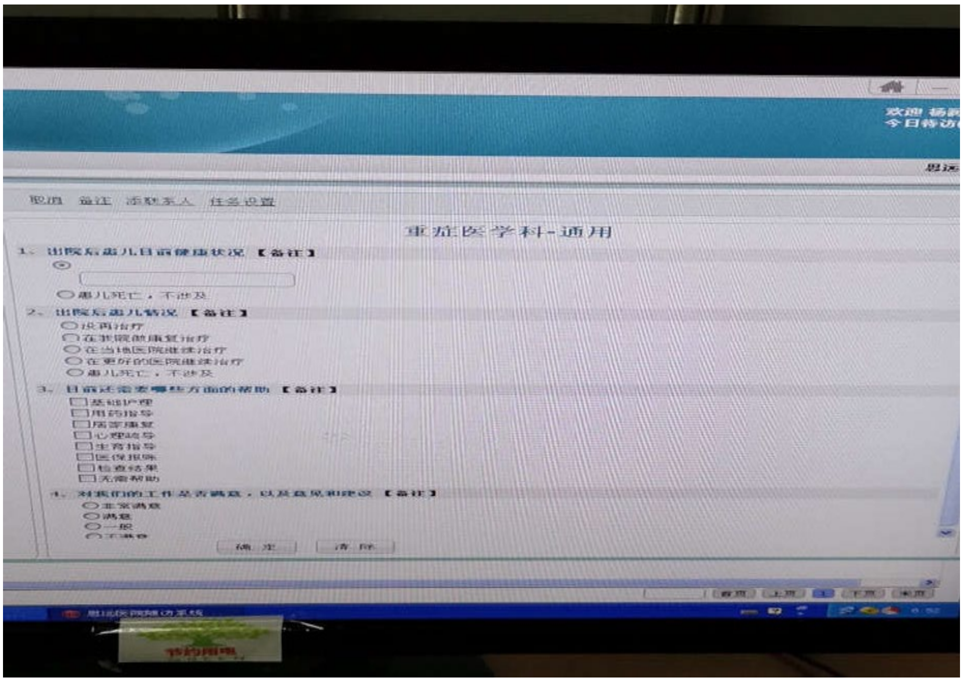

The reasons why we chose the four indicators

We selected the GV indices based on the previous kinds of literature and had a crude understanding of their strengths and weaknesses. SD, an indicator which was the most frequently used indicator in previous research (1)， does not address non-Gaussian skewed asymmetrical distribution or outliers. However, remains a fairly robust measure because a linear relation has been established between the interquartile range and the SD (2). There are many indicators based on SD, such as CV, an indicator that represents SD corrected for mean (1), is also a classic method to measure GV. It has the same limitations as SD (3) so that it couldn't replace SD. Considering the wide use of SD and being the basis for several indicators (CV, MAGE, J index), we finally included SD as a representative indicator that hasn't included time information. GLI (4) and MAG (5) considered the speed and magnitude of change and the time interval between glucose measurements. They both included the time information. Besides, the average consecutive absolute change percentage (ACACP) (6) was introduced, an index can be used in the real-time clinical decision conveniently. As a consequence, these four representative indicators (SD, MAG, GLI, and ACACP) were finally identified.

References

1. Siegelaar SE, Holleman F, Hoekstra JB, DeVries JH. Glucose variability; does it matter. Endocr Rev. 2010. 31(2): 171-82.

2. Rodbard D. Optimizing display, analysis, interpretation and utility of self-monitoring of blood glucose (SMBG) data for management of patients with diabetes. J Diabetes Sci Technol. 2007. 1(1): 62-71.

3. Rodbard D. Interpretation of continuous glucose monitoring data: glycemic variability and quality of glycemic control. Diabetes Technol Ther. 2009. 11 Suppl 1: S55-67.

4. Donati A, Damiani E, Domizi R, et al. Glycaemic variability, infections and mortality in a medical-surgical intensive care unit. Crit Care Resusc. 2014. 16(1): 13-23.

5. Hermanides J, Vriesendorp TM, Bosman RJ, Zandstra DF, Hoekstra JB, Devries JH. Glucose variability is associated with intensive care unit mortality. Crit Care Med. 2010. 38(3): 838-42.

6. Sadan O, Feng C, Vidakovic B, et al. Glucose Variability as Measured by Inter-measurement Percentage Change is Predictive of In-patient Mortality in Aneurysmal Subarachnoid Hemorrhage. Neurocrit Care. 2020. 33(2): 458-467.
